# Prediction of Inadequate Bowel Preparation Using Total and Segmental Colon Transit Time in Patients with Chronic Constipation: Some Different Outcomes

**DOI:** 10.1155/2019/2328054

**Published:** 2019-10-13

**Authors:** Chunying Zhai, Qiyang Huang, Ningli Chai, Wengang Zhang, Enqiang Linghu

**Affiliations:** ^1^Department of Gastroenterology, Chinese PLA General Hospital, Fuxing Road 28, Beijing, HaiDian District, China; ^2^Department of Gastroenterology, Beijing Puren Hospital, Chongwai Road 100, Beijing, DongCheng District, China

## Abstract

**Aims:**

Radio-opaque markers have been widely used in the study of colon motility in patients with chronic functional constipation (FC). Here, we evaluate the relationship between the colon transit time (CTT) and the Boston Bowel Preparation Scale (BBPS) to determine whether CTT is a sufficient predictor of bowel preparation in patients with chronic functional constipation.

**Methods:**

A total of fifty-six patients with constipation and fifty-two healthy controls (HC) were enrolled in this study. All subjects underwent the colonic transit study using radio-opaque markers and were given a follow-up colonoscopy examination on day 3 to 7 to determine BBPS. The correlation between total and segmental CTT and BBPS was evaluated, and risk factors for predicting inadequate bowel preparation were determined.

**Results:**

In our study, we found some distinct outcomes compared with previous studies. The mean total CTT (TCTT) was determined to be 43.37 ± 18.82 h in the FC group and 23.08 ± 10.18 h in the HC group. This difference was found to be significant for both the total and segmental CTTs between the two groups (*P* < 0.05). Further, TCTT was negatively correlated with BBPS both in the FC (*r* = −0.899, 95% CI -0.748 to -0.925, *P* < 0.001) and the HC (*r* = −0.978, 95% CI -0.854 to -1.003, *P* = 0.004) groups, as was segmental CTTs and segmental BBPS (*P* < 0.05). In the case of patients with slow transit constipation, multivariate logistic regression analysis indicated that prolonged TCTT (OR 0.722, 95% CI 0.589-0.885, *P* = 0.002) was independently associated with poor bowel preparation. The total and right to left CTTs were found to predict inadequate bowel preparation and exhibited the best sensitivity and specificity at 48.0 h, 15.5 h, 17.5 h, and 19.0 h, based on ROC curve analysis.

**Conclusions:**

The CTT test represents a valuable method for predicting the level of bowel preparation prior to a colonoscopy examination. That is, both total and segmental CTTs can be considered an objective predictor of bowel preparation prior to colonoscopy. The present study demonstrates some distinct results relative to previous studies, including STC subtype proportion in FC, the proportion of inadequate bowel preparation in the STC subtype, and the cut-off value of TCTT for predicting inadequate bowel preparation.

## 1. Introduction

Chronic functional constipation is a common gastrointestinal disorder, with a global prevalence of 12.0-17.0% [1]. Constipation not only significantly impairs quality of life but also poses an economic burden, with direct health-care costs reaching 7500 US dollars per patient annually and indirect costs that include a loss of productivity due to work absences [1, 2]. In patients with slow transit constipation, reduced bowel movements could lead to a less effective washout of laxatives, followed by inadequate bowel preparation. This hypothesis was recently confirmed by Park and colleagues [3]. Accordingly, a method to predict the level of bowel preparation in patients with chronic constipation would be beneficial for increasing colonoscopy success rate.

Colonic electric waves are suggested to be generated by at least four pacemakers, which are presumably located at the ileocecal junction, cecocolonic junction, mid-transverse colon, and colon sigmoid junctions. Shafik et al. postulated that colonic inertia might be a result of a pathological process of those pacemakers [4]. Further, another study demonstrated that the rectal sigmoid junction is the boundary separating the sigmoid colon and rectum, though this transition zone has different definitions [5]. In the present study, we used radio-opaque markers to evaluate total and segmental colonic transit time (CTT). The use of radio-opaque markers and abdominal X-ray is a standard approach for determining CTT [6]. Overall, this test measures the total and segmental transit time of the colon, provides objective information regarding abnormal bowel function, helps establish the appropriate treatment, and determines disease classification based on the pathophysiology of chronic constipation [7]. Furthermore, this test is commonly used to distinguish constipation subgroups, such as normal or slow transit times in patients with delayed total colon transit [8].

The quality of colon cleansing represents a key determinant of colonoscopy quality, as it is related to polyp detection rates, complete examinations, and the overall efficiency. The Boston Bowel Preparation Scale (BBPS) is a widely used and highly tractable bowel cleanliness scoring system [9]. A recent study by Heron and colleagues showed that higher scores (mean 6.1-7.1) of BBPS were significantly associated with the ability to detect lesions ≥ 5 mm, in comparison with inadequate bowel preparations (mean 4.5-5.1). In the current study, we aimed to evaluate the relationship between the total and segmental CTTs and BBPS to determine whether the CTT is sufficient to predict inadequate bowel preparation in patients with chronic constipation. To evaluate this question, we leveraged the experimental design described by Park et al. [3], including CTT examination and the BBPS system for evaluating bowel preparation. We enrolled a group of functional constipation patients, and we report distinct results regarding the relationship between CTT and bowel preparation in patients with chronic constipation.

## 2. Subjects and Methods

### 2.1. Ethical Approval

This study was approved by the Peking Puren Hospital, and informed consent was obtained from all of the patients prior to their enrolment in this study. All procedures performed in this study were in accordance with the ethical standards of the institutional and/or national research committee and with the *Helsinki Declaration* and its later amendments, or comparable ethical standards.

### 2.2. Subjects

The study was carried out prospectively. A total of fifty-six adults aged 18-80 years with functional constipation, who visited the hospital as outpatients from November 1, 2015, to November 1, 2017, were enrolled in this study according to the diagnosis standards of the Rome III criterion [10]. A two-week washout period was undertaken for patients receiving laxatives that might influence bowel habit. Participants were excluded based on the following criteria: (1) a history of abdominal and pelvic surgery, (2) a history of gastrointestinal cancer, diabetes, Parkinson's disease, thyroid disease, or IBD, and (3) a history of pregnancy in one year before the test. Fifty-two adults with a family history of gastrointestinal cancer who were scheduled for a colonoscopy examination at the same time were recruited for the CTT test and colonoscopy as the healthy control (HC) group. All subjects were receiving colonoscopies for routine screening purposes only, with no reported changes in bowel habits. We evaluated the symptoms of the control group based on the Wexner score, as shown in Table 1. Subjects were excluded if they had a significant current or previous medical history, were regularly taking medication (e.g., opioids, aluminized drugs, antidepressants, and calcium antagonists) that may affect the GI or central nervous system, or had donated blood within the past six months, or the Wexner score ≥ 2. The control group had habits of defecate frequency1-2 times/day or 1-2 days and never or seldom suffered from difficulty in defecate or a sense of defecation or defecation time longer than 10 minutes.

### 2.3. CTT Examination

A single capsule containing 24 radio-opaque markers (Sitzmarks, Konsyl Pharmaceutical, TX, USA) was taken once per day for three consecutive days at 8 am. The protocol was similar to that described by Bharucha and some authors and colleagues [11]. Simple abdominal radiographs were taken at 8 am on day four of the study. Markers were counted in three segments of the colon. The “imaginary lines” from the fifth lumbar vertebra to the left anterior superior iliac spine and to the right pelvic outlet were used as landmarks. The number of markers counted on the films was interpreted as the number of hours of transit for the whole and segment of the colon. Total colon transit time (TCTT) was calculated as the total number of markers in the colon, and segmental CTT was calculated as the number of markers in the three colonic segments, referred to as the right colon CTT (RTT), left colon CTT (LTT), and rectal sigmoid CTT (RSTT), as previously described by Miller and some authors and colleagues [12]. A mean TCTT of 30 h was chosen as the standard colon transit time, in accordance with previous studies [3, 13, 14]. Based on this standard, we classified FC patients with a TCTT of <30 h as the normal transit constipation (NTC) group and those with TCTT of ≥30 h as the slow transit constipation (STC) group. All subjects were asked to maintain their usual dietary habits and complete questionnaires regarding demography, the main symptoms (abdominal distension, abdominal pain, feeling of unfinished defecation, and prolonged defecating time ≥ 20 min), total bowel movements per week, and a three-day detailed diet diary for the duration of the study period.

### 2.4. BBPS Evaluation

All subjects who underwent a CTT test were given a follow-up colonoscopy examination three to seven days later. Subjects were instructed to take 4 L of PEG in two doses: 2 L of solution in the evening at 8 pm within one hour, and 2 L of solution the following morning at 5 am within one hour. A colonoscopy was performed within four to six hours following the bowel preparation. Colonic cleansing was scored according to the BBPS standard, as shown in Table 2 [15]. The total and segmental colon BBPS were scored separately by two endoscopy doctors. The left colon was defined as 20 cm before and after the splenic flexure, the right colon was defined as the upper segment, and the rectal sigmoid colon was defined as the lower segment. The total and segmental BBPS were averaged to obtain the final score. The minimum total score was 0, and the maximum total score was 9. Using a score of 6 as a reference, subjects who received ≥6 points were classified as having proper bowel preparation, and those who received <6 points in total, or <2 points in any segment, were classified as having inadequate bowel preparation [9, 15].

### 2.5. Statistical Analysis

All statistical analyses were implemented in SPSS 20.0. Data that are normally distributed are presented as mean ± SD or as proportions (%), and the data of BBPS that do not conform to the characteristics of normal distribution are presented as median with quartiles. Comparisons between the FC and the HC group were evaluated by unpaired Student's *t*-test for continuous data and by Chi-square test for nominal data and by Mann-Whitney test for abnormal distribution data. Pearson's correlation coefficient was used to determine the correlation between total and segmental CTTs and BBPS. Univariate and multivariate logistic regression was used to calculate the odds ratio (OR), with 95% confidence intervals (95% CI) for evaluating the risk of poor bowel preparation. Receiver operating characteristic (ROC) analysis was performed to calculate the cut-off value, sensitivity, specificity, and accuracy for discerning patients with inadequate bowel preparation. The optimal cut-off value was determined as the point that yields the best sensitivity and specificity on the ROC curve. A *P* value of less than 0.05 was considered statistically significant.

## 3. Results

### 3.1. Patients Characteristics

Fifty-six patients with functional constipation (22 males) and fifty-two healthy controls (26 males) were recruited. Demographic data from both groups are summarized in Table 3. No statistical differences in age, history of smoking, sex, BMI, or calorie intake were observed between the FC and HC groups.

### 3.2. CTT Measurement

The mean TCTT was determined to be 43.37 ± 18.82 h in the FC group and 23.08 ± 18.18 h in the HC group. The total and segmental CTTs (TCTT, RTT, LTT, and RSTT) in the two groups are shown in Table 4. This difference in total and segmental CTT was found to be statistically significant between the FC and HC groups (*P* < 0.05). Of the 56 constipation patients evaluated, 40 patients were classified in the STC group (71.43%), while 16 patients were classified in the NTC group (28.57%).

### 3.3. BBPS Evaluation

The mean BBPS was determined to be 6.5 (4.0-8.0) for the FC group, and 8.0 (6.0-9.0) for the HC group. The total and segmental BBPS values for both groups are given in Table 4. Overall, significant differences were observed both in the total, left colon, and rectal sigmoid colon BBPS between the FC and HC groups (*P* < 0.05). However, no significant differences in segmental BBPS were observed in the right colon between the FC and HC groups (*P* > 0.05). Of the 56 constipation patients evaluated in the FC group, 32 patients were found to have inadequate bowel preparation (57.14%), while in the HC group, 11 subjects had inadequate bowel preparation (21.15%). This difference was statistically significant (*χ*^2^ = 14.574, *P* < 0.001).

In the STC group, of the 40 patients evaluated, 26 patients were found to have inadequate bowel preparation (65.00%). This is in contrast to the NTC group, where only 5 subjects were found to have inadequate bowel preparation (31.25%), and this difference was statistically significant (*χ*^2^ = 5.268, *P* = 0.036). Further, the incidence of inadequate bowel preparation was determined to be similar between the NTC and HC groups (*χ*^2^ = 0.693, *P* = 0.405). Within the STC group, <3 bowel movements per week and prolonged TCTT were common in those determined to have inadequate bowel preparation (*P* < 0.05), as shown in Table 5.

### 3.4. Correlation between CTT and Bowel Preparation in Constipation Patients

In order to elucidate the relationship between CTT and bowel preparation, we determined the correlation between CTT and BBPS to evaluate whether CTT was sufficient to predict inadequate bowel preparation. Overall, CTT and BBPS were significantly negatively correlated in both the FC and HC groups. Further, both the total and segmental prolonged CTTs were related to inadequate bowel preparation. The correlation between CTT and BBPS is shown in Table 6.

**Figure 1:**
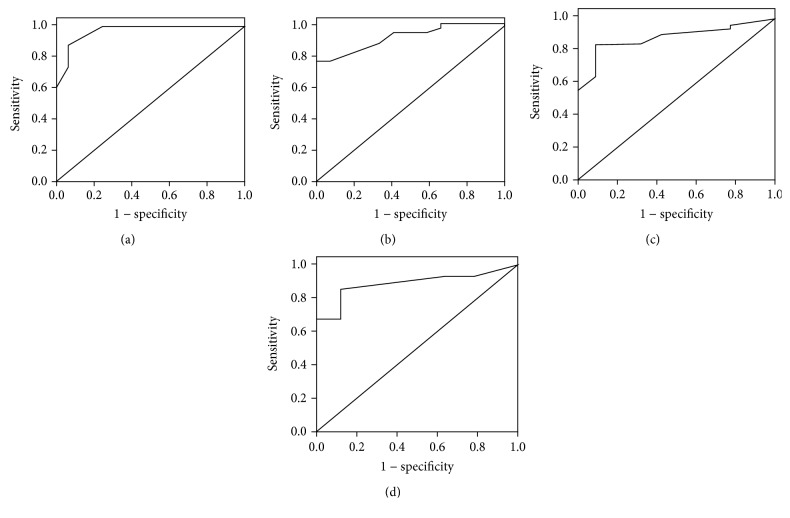
(a) A cut-off value of TCTT of 48.0 h predicts inadequate bowel preparation with 95.7% sensitivity and 85.4% specificity (AUROC = 0.969). (b) A cut-off value of RTT of 15.5 h predicts inadequate right colon preparation with 75.0% sensitivity and 85.7% specificity (AUROC = 0.866). (c) A cut-off value of LTT of 17.5 h predicts inadequate left colon preparation with 77.8% sensitivity and 75.0% specificity (AUROC = 0.818). (d) A cut-off value of RSTT of 19.0 h predicts inadequate rectal sigmoid colon preparation with 70.6% sensitivity and 91.9% specificity (AUROC = 0.893).

In the STC group, univariate logistic regression analysis indicated that bowel movements < 3/week (OR 4.750, *P* = 0.029) and TCTT > 48.0 h (OR 16.116, *P* < 0.001) were associated with poor bowel preparation, as shown in Table 7. We aimed at the above two significant indicators for further multivariate logistic regression analysis. However, the statistical outcome indicated that only TCTT (OR 0.722, 95% CI 0.589 to 0.885, *P* = 0.002) was independently associated with poor bowel preparation, after adjusting for age and gender, as shown in Table 8. Using the ROC curve analysis, we determined the cut-off value to predict inadequate bowel preparation in the STC group. We found that a cut-off TCTT of 48.0 h was able to predict inadequate bowel preparation with 95.7% and 85.4% sensitivity and specificity, respectively. We also determined that RTT of 15.5 h, LTT of 17.5 h, and RSTT of 19.0 h were able to predict related segmental inadequate bowel preparation (Figures 1(a)–1(d)) in the STC group and exhibited the optimal sensitivity and specificity. The diagnostic function of total and segmental CTT compared with BBPS for inadequate bowel preparation in the STC group is shown in Table 9.

## 4. Discussion

Recently, the colonic scintigraphy and the wireless motility capsule (WMC) test were validated for measuring whole gut transit and colon transit [16]. However, these methods to determine CTT were not widely adopted in clinical practices, due to its high cost and the existence of improved diagnostic techniques [17]. Although there are now several methods available for determining CTT, the method based on radio-opaque markers is considered to be the gold standard [18] and is widely used throughout the world.

In order to confirm the objectivity and practicality of the CTT test, we compared patients with functional constipation to a group of healthy controls. Data including age, sex, a history of smoking, BMI, GI symptoms, and a detailed three-day diet record were collected from all subjects, and no significant differences were found between the FC and HC groups. The primary findings from our study included that, in the FC group, the TCTT was 43.37 ± 18.82 h, and the segmental CTTs for the right, left, and rectal sigmoid colons were 12.44 ± 8.54 h, 15.78 ± 9.23 h, and 14.19 ± 10.17 h, respectively. For the FC group, the TCTT was determined to be 23.08 ± 10.18 h, and the segmental CTTs for the right, left, and rectal sigmoid colons were found to be 7.62 ± 5.73 h, 7.57 ± 5.41 h, and 7.84 ± 4.33 h, respectively. The difference in the total and segmental CTT between the FC and HC groups was found to be statistically significant (*P* < 0.05). That is, FC patients have a longer CTT compared with the HC group, and the CTT test had substantial value in the evaluation and identification of FC patients.

With regard to normal CTT, our results are supported by those of previous studies. For instance, Chan and colleagues [13] demonstrated that the mean total CTT in healthy Chinese adults was 24.5 h, which was very similar to the 23.08 h we observed in the HC group. Though a universal standard for CTT has not been established, 30 h is a commonly used benchmark for the standard colon transit time [11, 13, 14].

Using this standard of 30 h, we categorized a group of functional constipation patients in the STC and NTC groups, following Park et al. [3] However, we report some notably different results compared with previous studies. Of the 56 constipation patients, 40 were classified in the STC group (71.43%), while 16 patients were classified in the NTC group (28.57%). The proportion of the STC subtype was higher than the 51% reported by Park and colleagues [3] and the 42% reported by Shahid et al. [19]. These differences are likely attributed to a variety of factors. Firstly, the present study aimed to evaluate functional constipation patients, and patients with a history of hypertension, diabetes, cerebral disease, thyroid disease, surgical history, or chronic obstructive pulmonary disease were excluded. This is in contrast to previous studies, which did not exclude patients with these constipation-related conditions and may explain the reported discrepancies. Secondly, the present study used CTT of 30 h as the standard. However, in Shahid et al. [19], the authors used a standard CTT of 40 h. The longer CTT criterion may reduce the proportion of patients identified in the STC subtype. In the Park et al. study, the authors used not only a standard TCTT of 30 h but also 20 h as the mean right CTT and 10 h as the rectal sigmoid CTT to classify the STC subtype, which may also reduce the proportion of the identified STC patients. Finally, in this study, the small sample size and single hospital sourced subjects may have led to a decrease in representative samples and inflated the proportion of patients identified with the STC subtype. Overall, STC appears to be caused by impaired colonic motility and a dysfunctional enteric nervous system [20]. A recent study reported that some GI peptides, particularly neurotensin and motilin, are linked to impaired colonic motility in STC constipation [21].

Proper bowel preparation is an essential prerequisite for a successful colonoscopy examination. Factors known to be associated with inadequate bowel preparation include diabetes, cirrhosis, sex, history of stroke, and antidepressant use [22]. In this study, a BBPS < 6 was considered to be an inadequate bowel preparation, in accordance with previous studies. As such, subjects that received greater than 6 points were classified as having proper bowel preparation, and subjects that had less than 6 points in total, or less than 2 points for one of any three segments, were classified as having an inadequate bowel preparation [9]. The BBPS is a validated prep scoring system, with demonstrated consistency in both intra- and interobserver reliability. In this study, we evaluated bowel preparation using BBPS in the STC, NTC, and HC groups, and we observed inadequate bowel preparation in 65.00% of the STC group, in 31.25% of the NTC group, and in 21.15% of the HC group. In a recent study, inadequate bowel preparation was reported in 21.3% of all cases [9], a finding similar to the 21.15% we report in the healthy group in the present study. The proportion of inadequate bowel preparation was not significantly different between the NTC and HC groups. However, 65.00% of the patients in the STC group had poor bowel preparation, which was about two times higher than the 31.7% reported in Park et al. [3]. In accordance with a blind experimental design, the endoscopy doctors who scored the colonoscopy examination were not informed of the outcomes of the total and segmental CTT or the subject's group (i.e., FC or HC). Further, each subject's final BBPS was determined by averaging. This experimental design ensures the overall objectivity and reliability of the results. Another possible explanation for the higher proportion of inadequate bowel preparation in STC subtype reported in this study was the PEG used before the colonoscopy. PEG that is produced by different manufactures may have a different molecular weight, leading to changes in intestinal osmotic pressure that may affect the outcome of bowel preparation. Overall, the high proportion of inadequate bowel preparation observed in the STC patients illustrated that the STC patients may have different pathogenesis compared to the NTC patients.

In the case of patients with STC, CTT was significantly negatively correlated with BBPS in both the FC and the HC groups. That is, both the total and the segmental prolonged CTTs were found to be related to inadequate bowel preparation in our study. Total and right to left CTTs could be used to predict inadequate bowel preparation and showed the best sensitivity and specificity at 48.0 h, 15.5 h, 17.5 h, and 19.0 h based on the ROC curve analysis. The meaning of a cut-off value is the CTT testing result which predicts inadequate bowel preparation with best sensitivity and specificity. According to these outcomes, when we conducted CTT test before colonoscopy examination and arrived at the total and segmental CTTs, we could use these cut-off values to help us to identify the person at risk of poor bowel preparation. If the total CTT was longer than 48.0 h, then the patient would have the risk of poor bowel preparation and the BBPS have a chance of <6 which might lead to unsuccessful colonoscopy. Similarly, if the RTT was longer than 17.5 h, the patient would have the risk of poor right bowel preparation and the RBBPS have a chance of <2 which might lead to dissatisfaction observation of the right colon. But the cut-off values were obtained from the present study which was a small sample single-center research. The precise value of number for diagnosis still needs the large sample multicenter trials.

However, in Park et al. [3], the authors demonstrated that the best TCTT cut-off value was 37.0 h, which is substantially lower than the 48.0 h reported in the present study. The difference could be attributed to the following factors: Firstly, the subjects enrolled were different. In our study, a group of functional constipation patients were enrolled and STC subtype was evaluated. But in Park et al. [3], the enrolled chronic constipation patients included functional constipation and patients with a history of hypertension, diabetes, cerebral disease, thyroid disease, surgery, and chronic obstructive pulmonary disease. We demonstrated that the STC subtype has a longer CTT, so this difference in inclusion criteria may lead to a shorter CTT. Secondly, Park et al. did not report a cut-off value of the segmental CTTs for predicting segmental bowel preparation. Finally, we made some modifications when evaluating segmental BBPS, in order to be consistent with the segmental CTT. That is, the left colon was defined as 20 cm before and after splenic flexure, and the right and rectal sigmoid colons were defined as the upper segment and lower segment, respectively. This is the first report of this colon division method, which was implemented for consistency between studies of CTT and BBPS.

Constipation patients were found to be at risk for inadequate bowel preparation. Dong and colleagues previously demonstrated that infrequent bowel movements (<3/week) were associated with poor bowel preparation [23]. In this study, infrequent bowel movements (<3/week) also tended to be associated with poor bowel preparation, but this association was not statistically significant (OR 1.179, 95% CI 0.041-33.666, *P* = 0.923), after removing the influence of age and sex. Multivariate logistic regression analysis indicated that prolonged TCTT (OR 0.722, 95% CI 0.589-0.885, *P* = 0.002) was independently associated with poor bowel preparation. These findings demonstrate that CTT is a critical factor associated with bowel preparation. CTT measured prior to the colonoscopy could therefore be useful for the development of individualized strategies and objective methods for predicting bowel preparation. Some previous studies have demonstrated a few methods used to predict inadequate bowel preparation prior to colonoscopy. Hassan and colleagues reported a model for a combination of factors that can be used to predict bowel preparation. However, this method was found to be restricted by race and gender [24]. Fatima et al. reported that the description of the patient's last stool may be used to predict bowel preparation. However, this method depends entirely on the patient's personal description [25]. As such, there are currently few objective and efficient tools available to adequately predict bowel preparation quality prior to colonoscopy, especially in constipation patients needing additional interventions prior to the colonoscopy. Therefore, we aimed to identify measurable and accurate prediction methods for constipation patients who were most likely to experience inadequate bowel preparation. In this study, we determined that total and segmental CTT represent the best predictors of inadequate bowel preparation.

We note that there are some limitations to the current study. First, all subjects were recruited from a single medical center and the total number of subjects was relatively small (fifty-six patients and fifty-two controls). Second, in this study, the diagnosis of constipation was performed in accordance with the Rome diagnostic criteria, which relies on symptomatology. Therefore, it is possible that IBS-C was misdiagnosed as chronic constipation in some patients. Third, in some previous studies, CTT was assessed by counting the number of radio-opaque markers on a plain radiogram on day four and added to the number of radio-opaque markers counted on day seven. But in this study, we only counted the number on day four, which may lead to a relatively shorter CTT. In addition, the division of the colon segments was not completely consistent when segmental CTT and BBPS were evaluated. In future studies, these known confounding factors should be adjusted in order to reduce biases.

## 5. Conclusions

The CTT test represents a valuable method for predicting the level of bowel preparation prior to a colonoscopy examination. Despite the limitations associated with this test, this study was based on pacemaker theoretical foundation and demonstrated that total and segmental CTTs sufficiently predicted bowel preparation prior to colonoscopy. This study is in contrast to previous studies, with regard to STC subtype proportion in FC patients, the proportion of inadequate bowel preparation in STC subtype, and the cut-off value of TCTT for predicting inadequate bowel preparation. Further research is needed to fully evaluate these predictors of bowel preparation.

## Figures and Tables

**Table 1:** Wexner rating scale.

Defecate frequency	Score
1-2 times/1-2 days	0
2 times/week	1
Suffered from defecate	Score
Never	0
Seldom	1
Sense of unfinished defecation	Score
Never	0
Seldom	1
Pain	Score
Never	0
Seldom	1
Defecation time	Score
<5 min	0
5-10 min	1
Assist defecate	Score
No assist	0
Agent	1
Defecation failure	Score
Never	0
1-3/24 h	1
Medical history	Score
Without	0
<5 years	1

**Table 2:** Boston Bowel Preparation Scale.

Score	Colon condition
0	Unprepared colon segment with mucosa not seen due to solid stool that cannot be cleared.
1	Portion of mucosa of the colon segment seen, but other areas of the colon segment not well seen due to staining, residual stool, and/or opaque liquid.
2	Minor amount of residual staining, small fragments of stool, and/or opaque liquid, but mucosa of colon segment seen well.
3	Entire mucosa of colon segment seen well with no residual staining, small fragments of stool, or opaque liquid.

**Table 3:** Main baseline features of the enrolled subjects.

Features	FC (*n* = 56)	HC (*n* = 52)	*P*
Age (years)	62.3 ± 16.5	58.8 ± 15.7	0.375
Males	22 (39.3%)	26 (50.0%)	0.423
BMI (kg/m^2^)	23.4 ± 2.8	22.3 ± 2.3	0.831
History of smoking	18(32.1%)	22(42.3%)	0.089
Symptoms			
Distension	35(62.50%)	0	
Pain	14(25.00%)	0	
Unfinished defecation	16(28.50%)	2(3.84%)	<0.001
≥20 min	19(33.92%)	0	
Bowel movements			
≥3/week	11(19.64%)	50(96.15%)	<0.001
1-3/week	38(67.85%)	2(3.84%)	<0.001
≤1/week	7(12.51%)	0	
Calorie intake (kcal)	1718.6 ± 535.4	1809.5 ± 487.8	0.362
Protein (g)	78.4 ± 31.6	82.3 ± 35.3	0.257
Carbohydrate (g)	239.7 ± 98.5	247.9 ± 103.8	0.764
Fat (g)	61.6 ± 26.5	68.3 ± 22.6	0.525

**Table 4:** Total and segmental CTTs and BBPS in two groups.

	CTT (h)				BBPS			
	FC (*n* = 56)	HC (*n* = 52)	*t*	*P*	FC (*n* = 56)	HC (*n* = 52)	*Z*	*P*
R	12.44 ± 8.54	7.62 ± 5.73	3.426	0.004	2.0 (1.0-3.0)	2.0 (1.0-3.0)	-0.973	0.330
L	15.78 ± 9.23	7.57 ± 5.41	5.579	<0.001	2.0 (1.0-3.0)	3.0 (2.0-3.0)	-2.613	0.009
RS	15.19 ± 10.17	7.84 ± 4.33	4.822	<0.001	2.0 (1.0-3.0)	3.0 (2.0-3.0)	-2.212	0.027
Total	43.37 ± 18.82	23.08 ± 10.18	4.357	<0.001	6.5 (4.0-8.0)	8.0 (6.0-9.0)	-2.380	0.017

CTT: colon transit time; BBPS: Boston Bowel Preparation Scale; R: right colon; L: left colon; RS: rectal sigmoid colon; Total: total colon.

**Table 5:** Characteristics compared between adequate and inadequate bowel preparation in the STC group.

Features	Total (*n* = 40)	Adequate (*n* = 14)	Inadequate (*n* = 26)	*P*
Age (years)	61.20 ± 15.26	63.50 ± 15.30	60.21 ± 15.41	0.540
Males	18 (45.00%)	6 (42.85%)	12 (46.15%)	0.842
BMI (kg/m^2^)	23.40 ± 2.47	23.96 ± 2.10	23.16 ± 2.61	0.362
History of smoking	18 (45.00%)	6 (42.85%)	12 (46.15%)	0.973
Symptoms				
Distension	29 (72.50%)	9 (64.29%)	20 (76.92%)	0.393
Pain	10 (25.00%)	3 (21.42%)	7 (26.92%)	0.702
Unfinished defecation	10 (25.00%)	2 (14.28%)	8 (30.76%)	0.251
≥20 min	16 (40.00%)	4 (25.00%)	12 (46.15%)	0.257
Bowel movements				
<3/week	33 (82.50%)	9 (64.28%)	24(92.31%)	0.026
TCTT (h)	45.23 ± 15.24	39.50 ± 6.25	58.71 ± 9.21	<0.001

STC: slow transit constipation.

**Table 6:** Pearson's correlation between CTT and BBPS in two groups.

	*r*	95% CI	*P*
Right colon			
FC	-0.788	-0.635~-0.862	0.003
HC	-0.813	-0.726~-0.874	<0.001
Left colon			
FC	-0.772	-0.627~-9.746	<0.001
HC	-0.804	-0.752~-1.153	<0.001
Rectal sigmoid			
FC	-0.781	-0.693~-0.847	<0.001
HC	-0.737	-0.563~-0.863	<0.001
Total colon			
FC	-0.899	-0.748~-0.925	<0.001
HC	-0.978	-0.854~-1.003	0.004

*r*: correlation coefficient.

**Table 7:** Univariate logistic regression analysis for inadequate bowel preparation in the STC group.

Features	OR	*P*
Age (years)		
>70	0.459	0.453
Male	0.077	0.781
Smoking	0.077	0.781
BMI (kg/m^2^)		
>25	<0.001	1.000
Symptoms		
Distension	0.860	0.354
Pain	<0.001	1.000
Unfinished defecation	1.429	0.232
≥20 min	0.045	0.833
Bowel movements		
<3/week	4.750	0.029
TCTT (h)		
>48	16.116	<0.001

OR: odds ratio; TCTT: total colon transit time.

**Table 8:** Multivariate logistic regression analysis for inadequate bowel preparation in the STC group.

Features	Wals	OR	95% CI	*P*
Age	2.149	1.129	0.960-1.328	0.143
Sex	0.884	2.422	0.109-53.638	0.576
<3/week	0.66	1.935	0.090-41.944	0.674
TCTT (h)	5.970	0.631	0.434-0.917	0.016
Removal of age and gender				
<3/week	0.009	1.179	0.041-33.666	0.923
TCTT (h)	9.819	0.722	0.589-0.885	0.002

STC: slow transit constipation; OR: odds ratio; TCTT: total colon transit time.

**Table 9:** Diagnostic function of total and segmental CTTs compared with BBPS for inadequate bowel preparation in the STC group.

TCTT (48.0 h)	BBPS		RTT (15.5 h)	RBBPS		LTT (17.5 h)	LBBPS		RSTT (19.0 h)	RSBBPS	
	<6	≥6		<2	≥2		<2	≥2		<2	≥2
≥48.0	20	3	≥15.5	14	4	≥17.5	16	5	≥19.0	14	2
<48.0	1	16	<15.5	6	16	<17.5	4	15	<19.0	3	21
Total	21	19	Total	20	20	Total	20	20	Total	17	23

TCTT: total colon transit time; BBPS: Boston Bowel Preparation Scale; RTT: right colon transit time; RBBPS: right colon Boston Bowel Preparation Scale; LTT: left colon transit time; LBBPS: left colon Boston Bowel Preparation Scale; RSTT: rectal sigmoid colon transit time; RSBBPS: rectal sigmoid colon Boston Bowel Preparation Scale.

## Data Availability

The authors can provide the underlying data for publication if necessary.
